# Resisting Influence: How the Strength of Predispositions to Resist Control Can Change Strategies for Optimal Opinion Control in the Voter Model

**DOI:** 10.3389/frobt.2018.00034

**Published:** 2018-04-17

**Authors:** Markus Brede, Valerio Restocchi, Sebastian Stein

**Affiliations:** ^1^Agents, Interactions, and Complexity Group, ECS, University of Southampton, Southampton, United Kingdom; ^2^Institute of Life Sciences, University of Southampton, Southampton, United Kingdom; ^3^Southampton Business School, University of Southampton, Southampton, United Kingdom

**Keywords:** opinion control, voter dynamics, scale-free networks, optimization, influence maximization

## Abstract

In this paper, we investigate influence maximization, or optimal opinion control, in a modified version of the two-state voter dynamics in which a native state and a controlled or influenced state are accounted for. We include agent predispositions to resist influence in the form of a probability *q* with which agents spontaneously switch back to the native state when in the controlled state. We argue that in contrast to the original voter model, optimal control in this setting depends on *q*: For low strength of predispositions *q*, optimal control should focus on hub nodes, but for large *q*, optimal control can be achieved by focusing on the lowest degree nodes. We investigate this transition between hub and low-degree node control for heterogeneous undirected networks and give analytical and numerical arguments for the existence of two control regimes.

## 1 Introduction

Processes of opinion formation play a role in a variety of real-world problems, ranging from political elections to marketing and product adoption, see Castellano et al. (2009), Sîrbu et al. (2017) for recent reviews. Very often, these processes involve peer-to-peer interaction (Easley and Kleinberg, 2010) and thus take place on social networks. In this context, the natural question arises how an external party with a certain amount of resources at its disposal can steer such a social system in a desired direction, maybe either with the intent of maximizing the adoption of products (Kempe et al., 2003; Bharathi et al., 2007, 2010; Goyal et al., 2014) or for the purposes of political influence in the so-called campaign problem (Hegselmann et al., 2015).

Starting with the seminal study of Kempe et al. (2003) on influence maximization, work in this area has strongly focused on the independent cascade model or related versions of threshold models, which have been studied in competitive and non-competitive settings (Kempe et al., 2003; Bharathi et al., 2007, 2010; Goyal et al., 2014). In the independent cascade model, influencing parties strategically distribute seeds, which can then cause cascades of influence spread. However, while allowing for neat solutions using optimal percolation (Morone and Makse, 2015), in the independent cascade model, agent behavior is assumed to be fixed once committed to a certain opinion, thus not allowing for dynamical change subject to competing internal or external influence over time. Models of this type thus appear not suitable for a range of applications (Kuhlman et al., 2013) in which the interest is in dynamic opinion change.

Recognizing this limitation of the independent cascade model, recent work has also started to consider opinion control in dynamic models of binary opinion change, which appear more suitable to capture dynamic phenomena of opinion change if agents don’t have strong commitment to decisions. Research in this area so far has considered models based on the kinetic Ising model (Liu and Shakkottai, 2010; Laciana and Rovere, 2011; Lynn and Lee, 2016), a variant of the AB model (Arendt and Blaha, 2015), which results in majority-like dynamics, and the voter dynamics (Kuhlman et al., 2013; Yildiz et al., 2013; Masuda, 2015). Whereas in the kinetic Ising model, agents change opinions according to a majority-like dynamics, in the voting dynamics agents adopt opinions of randomly selected neighbors (Clifford and Sudbury, 1973; Holley and Liggett, 1975). In contrast to, e.g., the Glauber dynamics underlying the kinetic Ising model, opinion changes of agents in the voting dynamics are caused by the pressure of the majority of their neighbors only in an averaged sense and the state of the majority does not play a direct role when making updating decisions. With differing effects of majority pressure, one thus finds differences in model behavior (Castellano et al., 2009). Nevertheless, of interest for our study below, for the kinetic Ising model, recent work has pointed out that optimal influence allocations may shift from focusing at high-degree nodes to low degree nodes depending on the *social* temperature of opinion change (Lynn and Lee, 2016). The work of Lynn and Lee (2016) demonstrates that hub control may not be optimal for all types of social contagion processes and hub nodes may play different roles at different stages of the dynamics (Quax et al., 2013). However, the focus of the present study is on the voting dynamics. In this context, Mobilia was first to investigate the impact of an agent favoring one opinion, a so-called “zealot” (Mobilia, 2003; Mobilia and Georgiev, 2005), which was later extended to considerations of inflexible voters (Mobilia et al., 2007). Zealots, or partisan voters, can be interpreted as external influence on the system. Whereas in the voting dynamics consensus is typically reached (Castellano et al., 2009), the mutual presence of multiple opposing zealots can lead to the co-existence of different opinions in equilibrium (Mobilia and Georgiev, 2005; Mobilia et al., 2007). Effects of *zealotry* in the voting dynamics are of considerable interest in the literature and have been studied in various settings. For instance, considerations of error-prone zealots have been addressed in Masuda et al. (2010), Masuda and Redner (2011). Further recent studies include voter models with a large number of states (Waagen et al., 2015), extensions to the non-linear q-voter model (Mobilia, 2015), an exploration of the role of mass media in multi-state voter models (Hu and Zhu, 2017), or, more recently, a study on the role of noise in the mean-field voter model with zealots (Khalil et al., 2018). However, none of the latter studies consider the role of strategically placed zealots.

In the context of opinion control in the voter model (Kuhlman et al., 2013) investigated control strategies focused on the highest-degree nodes, attempting to minimize control costs to achieve given threshold opinion shares. In other related work, Yildiz et al. (2013) proposed a new algorithm to find optimal control strategies, but mainly focused on the evaluation of the algorithm. Closest to the present work is the study of Masuda (2015), in which methods from linear algebra are used to explore optimal opinion control in the voter model. Masuda (2015) analyzes steady-state solutions of the master equation and then carries out numerical optimization to investigate optimal control strategies for artificially generated scale-free Barabási–Albert networks (Albert and Barabási, 2002) and a range of empirical social networks, including email-communication, co-authorship, and directed online social networks, finding that control protocols that focus on the highest-degree nodes are generally successful in heterogeneous undirected networks, but not necessarily in the case of directed networks. Findings of previous work on opinion control in the voter model thus seem to generally agree that optimal control on undirected social networks should generally be focused but not exclusively be concentrated on high-degree nodes (Kuhlman et al., 2013; Masuda, 2015). However, while proposing new algorithms and proving analysis of optimal opinion control for certain network topologies, up to our best knowledge, no previous study has investigated the role of the strength of predispositions to resist change on strategies for optimal opinion control.

Studies like Masuda (2015) have assumed the presence of two external influencing parties and investigated optimal strategies of an active optimizer competing against a passive strategy that does not actively pursue optimal control. Here, we propose a slightly different variant of the voter model, which may be closer in spirit to the independent cascade model but still allows for dynamic change of opinions. Instead of assuming the presence of a passive party, we consider a setting in which an active party attempts to align the system toward a goal, but agents are “fickle” in the sense that they might also spontaneously revert to the uninfluenced state with some probability. The inclusion of such fickleness allows us to study the dependence of opinion control on the strength of predispositions of agents to resist change. As we shall argue below, optimal control strategies are indeed very different in low and high predisposition settings on undirected networks, pointing out that previous findings like those of Kuhlman et al. (2013) and Masuda (2015) might not apply in all settings.

With the inclusion of predispositions, we aim to provide a framework that agrees with empirical evidence from recent work on social networks, in which it was observed that influence propagation follows a *complex contagion dynamics* (Centola and Macy, 2007; Centola et al., 2007; Centola, 2010; Hill et al., 2010; Romero et al., 2011). Complex contagion describes a process whereby repeated exposure is required for the adoption of opinions, behavioral patterns, products, etc. Such a process is enhanced by communities, in which individuals are repeatedly exposed to the same ideas. This contrasts with *simple contagion* (modeled, for example, by independent cascades), in which similarly to disease spreading, only one contact is required to spread a message. An immediate consequence of such different dynamics is that hubs typically represent the best influencer under a simple contagion dynamics, whereas targeting low-degree nodes may yield a larger spread for complex contagion (Alshamsi et al., 2017). By including predispositions to resist change in our model nodes can spontaneously revert to the uninfluenced state. Therefore, the proposed model reflects the repeated exposure needed in complex contagion to influence a node with high probability. Alshamsi et al. (2017) has recently shown that it may be best to influence low-degree nodes in complex contagion in a setting in which nodes are committed to a state once adopted. Our results complement these findings in dynamic settings and show further conditions under which it is best to target low-degree nodes instead of hub nodes.

Our study is organized as follows. In Section 2, we give a detailed description of the model employed and describe analytical and numerical methods to find optimal control strategies. Section 3 then gives our main findings and we finish with a summary and discussion in Section 4.

## 2 Materials and Methods

In the following, we consider a variant of the voter model (Clifford and Sudbury, 1973; Holley and Liggett, 1975) that accounts for spontaneous changes of opinions with a probability *q*. Let there be *N* agents with binary states *s_i_* = 1 or *s_i_* = 0, *i* = 1,…, *N*, which are connected by an unweighted social network given by its adjacency matrix $A={\left(a_{\mathrm{ij}}\right)}_{i,j=1}^{N}$. We consider undirected connections, hence *a_ij_* = *a_ji_* = 1 if there is a link between *i* and *j* and *a_ij_* = *a_ji_* = 0 otherwise. Additionally, we consider an external controller with opinion *s* = 1 which aims to align the system to its opinion. Control is exerted through the presence of additional in-neighbors with *s* = 1, i.e., a controlled node has an enhanced likelihood of choosing a neighbor with state *s* = 1 when updating. The controller thus influences the system through unidirectional links given by a vector $\vec{p}=\left(p_{1},\ldots ,p_{N}\right)$ where *p_i_* = 1 if the controller influences node *i* and *p_i_* = 0 otherwise. Without loss of generality, we assume that *s* = 1 is the desired state into which the controller wants to guide the system. However, “convinced” agents in state *s* = 1 may spontaneously revert to state *s* = 0.

In more detail, after random initialization of voters, the dynamics of opinions are updated as follows: (i) a focus agent *x* is picked at random, (ii) with probability (1 − *q*) agent *x* randomly selects one of its in-neighbors *y* and adopts the opinion of *y*, i.e., *s_x_* = *s_y_*. In the opposite case, i.e., with probability *q*, if in state *s* = 1 agent *x* will spontaneously revert to state *s* = 0. Steps (i) and (ii) are repeated until an equilibrium is reached.

The above process allows for analytical solutions. Define *u_i_* as the probability that node *i* will be in state *s* = 1. We can then write down the master equation

(1) $${\dot{u}}_{i}=\left(1-q\right)/\Sigma_{i}\;\left(\;\left(1-u_{i}\right)\;\left(\;\sum_{j}a_{\mathrm{ji}}u_{j}+p_{i}\;\right)-u_{i}\sum_{j}a_{\mathrm{ji}}\left(1-u_{j}\right)\;\right)-qu_{i},$$

where

(2) $$\Sigma_{i}=\sum_{j}a_{\mathrm{ji}}+p_{i}$$

is the in-strength or the sum of influences node *i* experiences. The first term in equation (1) captures the typical copying dynamics of the voter model, which occurs with probability 1-*q* [see, e.g., Masuda (2015)], and the second term −*qu_i_* accounts for spontaneous flips back into the uncontrolled state.

Equilibrium states can be obtained from

(3) $$\left(\text{diag}\left(\Sigma_{i}\right)-\left(1-q\right)A\right){\vec{u}}^{*}=\left(1-q\right)\vec{p},$$

where ${\vec{u}}^{*}=\left(u_{1}^{*},\ldots ,u_{N}^{*}\right)$ denotes the vector of equilibrium probabilities and diag(Σ*_i_*) stands for a diagonal matrix *D* with entries *D_ii_* = Σ*_i_* (cf. Appendix A for more detail). Again, following Masuda (2015), we next note that equation (3) gives a linear system, which is diagonally dominant for all *q*. Thus, an efficient way of solving equation (4) is by Jacobi iteration, where we start with $u_{i}^{\left(0\right)}=1/2$, *i* = 1,…, *N* and then iterate

(4) $$u_{i}^{\left(n+1\right)}=\left(1-q\right)/\Sigma_{i}\left(p_{i}+\sum_{j}a_{\mathrm{ji}}u_{j}^{\left(n\right)}\right),$$

where superscripts indicate the iteration number. Stationary solutions ${\vec{u}}^{*}$ then allow to estimate the share of votes influenced by the controller via $X=1/N\sum_{i}\;u_{i}^{*}$.

From equation (3), we can also read the mean-field solution for the controlled vote share when controllers are allocated randomly on an all-to-all connected network, finding

(5) $$X=\frac{1-q}{\rho +q}\rho ,$$

where $\rho =1/N\sum_{i}\;p_{i}$ is the density of controlled voters. It is straightforward to see the limiting cases of *q* = 0 and *q* = 1 in equation (5) corresponding to a perfectly controlled system (*X* = 1) and an uncontrollable system (*X* = 0), respectively. We can thus see that predisposition to resist change in the form of the flipping probability *q* quantify how difficult it is to control the system.

In the following, we are interested in optimal control strategies (as quantified by $\vec{p}$) for the external controller for given networks. As a model for social networks, we construct networks with power-law degree distributions *P*(*k*) ∝ *k*^−*α*^ according to the configuration model (Newman, 2010). For given control resource, $n=\sum_{i}\;p_{i}$ controls $\vec{p}$ are then first assigned randomly and then optimized using a stochastic hill climber. More precisely, we iterate the following scheme: (i) select a controlled node *x* and a yet uncontrolled node *y* at random, (ii) rewire the control from *x* (i.e., *p_x_* = 1, *p_y_* = 0) to *y* (i.e., *p_x_* = 0, *p_y_* = 1) if *X*(*p_x_* = 1, *p_y_* = 0) ≤ *X*(*p_x_* = 0, *p_y_* = 1). Optimization using steps (i) and (ii) is stopped once no rewiring of controls has been accepted for a certain number *T* of attempts and three different initial control allocations are explored to reduce the probability of ending up in local optima with stochastic hill-climbing. For network sizes of *N* = 1,000 nodes/voters that we shall investigate below, we typically set *T* = 10^4^, which makes sure no substantial improvements in control can be found any more. If not mentioned otherwise, we set *α* = 3 and run experiments with connectivity ⟨*k*⟩ = 3. In the following, we will explore the dependence of optimal opinion control strategies on the predisposition parameter *q* for various resource allocations *n* to the controller.

## 3 Results

In this section, we present our main findings. We start by outlining numerical results in Section 3.1 and then analyze two toy models, i.e., star networks and chains in Section 3.2. Exact solutions for the toy models illustrate the main claim of the paper and give analytical insight into the shift from optimal high- to low-degree control.

### 3.1 Numerical Results

**Figure 1:**
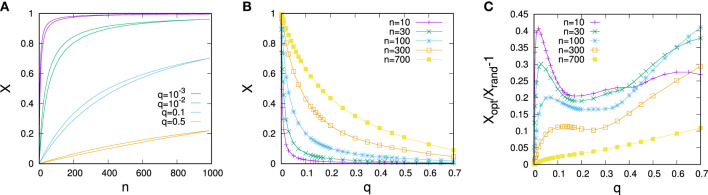
**(A)** Dependence of the controlled share of votes *X* on the resource of the controller *n*. For each color, the lower curve gives the vote share for random allocation of control and the upper curve vote shares for optimized allocations. **(B)** Dependence of optimized vote shares on the predisposition parameter *q* for various control resource endowments *n*. **(C)** Optimization gain relative to random control allocation for the scenario shown in **(B)**. The data are for networks composed of *N* = 1,000 nodes constructed for *α* = 3 and each data point represents an average over 50 randomly sampled network configurations. Error bars are of the size of the lines/points.

In Figure 1, simulation results on optimal vote control for scale-free networks of size *N* = 1,000 constructed for a scaling exponent *α* = 3 are visualized. Figure 1A compares the dependence of optimal vote shares and average vote shares under random allocation of control on the controller’s resource endowment *n* for various predisposition strengths *q*. As one would expect, the larger the resource endowment *n* and the lower *q*, the larger the share of controlled votes. Panel Figure 1B gives a further illustration of related experiments in which the optimal controller’s resource endowment was fixed, but the magnitude of *q* systematically varied. We again see that larger resource levels allow for tighter control, but the effects of control decline strongly with *q*. One notes that optimal placement of control can considerably improve vote shares relative to random allocation (Figure 1C), but absolute improvements due to optimization are very limited when either *q* or *n* are large. Maximum gains achievable by optimization starting from random allocations tend to be around 40–50% of the initial vote share.

**Figure 2:**
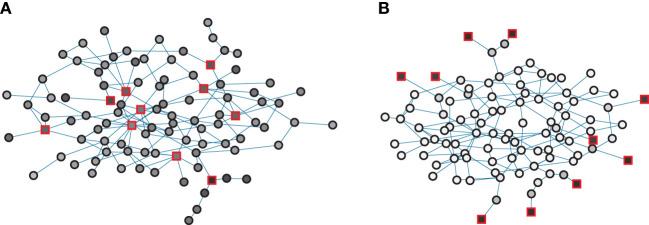
Examples of optimized control for a network of *N* = 100 nodes and *L* = 288 links for low *q* = 0.01 (panel **A**) and high *q* = 0.5 (panel **B**). The networks are constructed via a configuration model with *P*(*k*) ∝ *k*^−*α*^ with *α* = 3. Red boxes indicate controlled nodes, circles indicate ordinary nodes. Interior colors give relative control on a sliding scale from white (weakest control) to black (strongest control). The average opinion is ⟨s⟩ = 0.72 (panel **A**) and ⟨s⟩ = 0.034 (panel **B**).

What are the best resource allocations? We proceed by investigating the dependence of optimal control strategies on the strength of predispositions *q*. Figure 2 gives an illustration of some first results for a small network of *N* = 100 nodes where control was evolved for situations of low (panel A) and high (panel B) predisposition strength for a controller, which can influence 10 nodes. In the figure, controlled nodes are indicated by red boxes and the shading of nodes gives their average opinion state *u* for the chosen control scheme. Prevailing dark colors of nodes make it immediately obvious that the network can be strongly influenced in the low predisposition regime visualized in Figure 2A but largely resists control in the high predisposition regime in Figure 2B in which light colors dominate.

For a more systematic investigation, we define the average degree of a controlled node

(6) $$k_{\text{controlled}}=\frac{\sum_{i}\;p_{i}k_{i}}{\sum_{i}\;p_{i}},$$

where $k_{i}=\sum_{j}\;a_{\mathrm{ij}}$ is the degree of node *i*. Similarly, we also measure the standard deviation (SD)

(7) $$\sigma_{\text{k},\text{ controlled}}^{2}=\frac{\sum_{i}\;p_{i}{\left(k_{i}-k_{\text{controlled}}\right)}^{2}}{\sum_{i}\;p_{i}}.$$

of the distribution of controlled node degrees. To gain further insights about the dependence of control on degree, we also estimate likelihoods *P*_k, controlled_ of nodes to be controlled depending on their degrees.

**Figure 3:**
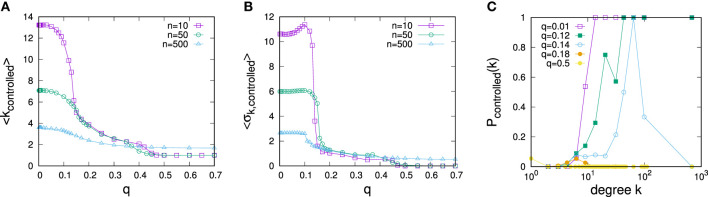
Dependence of optimal strategies for opinion control on the predisposition parameter *q*: **(A)** average degree of controlled nodes, **(B)** degree variance of controlled nodes. **(C)** Dependence of the probability of a node to be controlled on degree for control optimized for different values of *q* for *n* = 10. The data are for networks of *N* = 1,000 nodes with *α* = 3 and ⟨*k*⟩ = 3 and in **(A,B)**, each data point represents an average over 50 samples.

The dependence of all three measures on *q* are plotted in Figures 3A–C for various resource endowments *n*. As also seen in the example above, the figure suggests the existence of two control regimes. For small *q*, control is clearly focused on the highest degree nodes. The smaller *n*, the larger the average degree of the *n* highest degree nodes, and accordingly, we see relatively lower average degrees of controlled nodes the larger *n*. In contrast, for large *q*, control is clearly focused on low-degree nodes. In fact, as we see in the plot of the dependence of SDs of degrees of controlled nodes vs. the strengths of predispositions *q*, there is a sharp transition between the two control regimes, cf. Figure 3B. Starting from low *q* up to some critical point in *q*, the largest degree nodes are controlled in every instance, but control gradually includes more and more low-degree nodes (see Figure 3C). Beyond this point, control suddenly excludes the largest degree nodes and focuses on a mixture of low-degree nodes before eventually becoming firmly fixed on low-degree nodes for large *q*.

**Figure 4:**
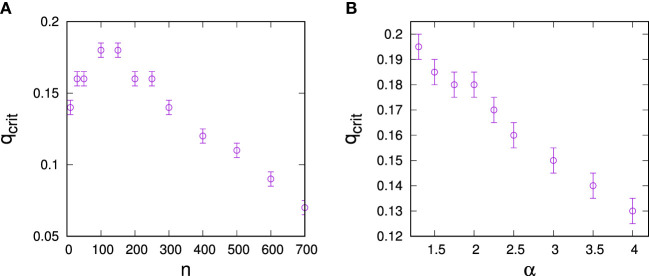
Dependence of the critical predisposition strength at which optimal control switches from hub control to low-degree control on **(A)** the total resource endowment of the controller *n*, **(B)** network heterogeneity for resource endowment *n* = 10 for the controller. The data are for networks of *N* = 1,000 with connectivity ⟨*k*⟩ = 3 and error bars result from the discretization of q-values when constructing σ_k, controlled(*q*)_ plots.

The low–high SD regime threshold depends on resource endowments. To evaluate this dependence, we have measured *q*-dependencies of ⟨σ_k, controlled_⟩ for various resource endowments *n* and determined critical points from the sharp transitions in the respective ⟨σ_k, controlled_⟩(*q*) plots. Results are illustrated in Figure 4A, where we see that thresholds between the regimes initially grow with *n*, then saturate, and decline.

We also investigated dependencies of thresholds on the structure of the social network to be controlled as quantified by the degree exponent *α*. For this purpose, we constructed configuration type models with fixed numbers of links for a range of *α*-parameters and again estimated critical points from the respective ⟨σ_k, controlled_⟩(*q*) plots. Results are shown in Figure 4B, where we see that more degree heterogeneous networks generally support a larger high-degree control regime.

**Figure 5:**
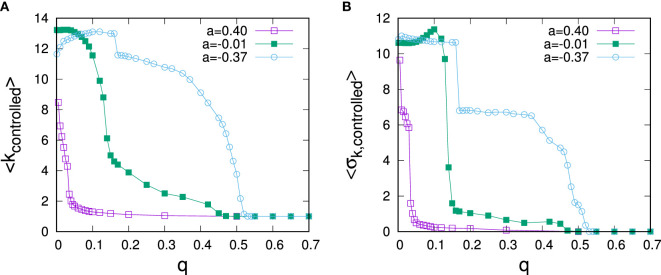
Dependence of average controlled degree **(A)** and SD of controlled degrees **(B)** on the predisposition to resist for social networks of different assortativity. The data are for networks of *N* = 1,000 nodes with *α* = 3 and ⟨k⟩ = 3 and data points represents averages over 50 runs.

All of the experiments conducted above have been carried out for networks with given degree heterogeneity, but without higher order correlations such as clustering or assortativity, which are typical for real-world networks (Newman, 2010). Because of the observed strong dependence of optimal control on degree, the impact of degree-mixing patterns on the optimal control allocation appears of particular interest. To address this question, we have constructed synthetic scale-free networks with dis-assortative and assortative degree mixing patterns. Such networks can be generated by starting from a neutrally assortative network and then randomly picking two connected pairs of nodes, ordering the nodes by degree, and rewiring to change connections toward linking the pair of nodes with highest and the pair with lowest degree (for increased assortativity) or re-linking nodes with largest degree differences (for dis-assortative mixing). Rewiring according to this scheme preserves the overall degree sequence and allows to tune degree mixing (Xulvi-Brunet and Sokolov, 2005). To investigate the role of degree mixing on control schemes, we have carried out rewiring to tune assortativity until no further reconnection moves could be carried out, resulting in networks with very strong dis-assortative and assortative degree mixing with *a* = −0.37 and *a* = 0.40 measured by Newman’s assortativity coefficient (Newman, 2003). Results for optimal control allocations for such networks are shown in Figure 5. It becomes apparent that assortativity has a strong influence on optimal control: Whereas the regime of hub control is strongly reduced for assortative networks, it is considerably extended for the case of dis-assortative degree mixing. As we shall see below, for *q* > 0, nodes are the more difficult to control, the larger their degree. Thus, in an assortatively mixed network, hub nodes tend to be surrounded by nodes, which are difficult to control, making it even more difficult to control the hub node itself. The effect results in a much lowered threshold for *q* at which periphery control becomes optimal. The contrary argument applies for disassortative networks. In this case, hub nodes are surrounded by nodes that can be more easily controlled, which, in turn, makes them easier to control even at large *q*, resulting in an extension of the regime of optimal hub control.

### 3.2 A Model of Star Networks and Chains

**Figure 6:**
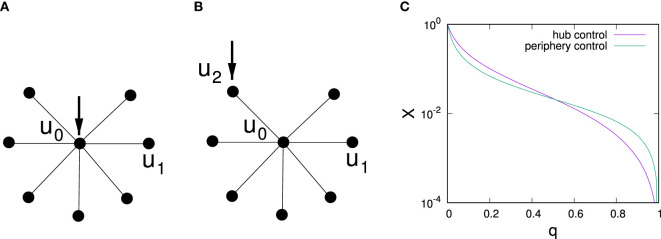
Illustration of a star network with control targeted at a central hub **(A)** and control targeted at a periphery node **(B)** and dependence of the average stationary vote shares for both scenarios on *q* **(C)** for a star network with one central hub and *k* = 15 spokes. Average probabilities of being in the controlled state are labeled *u*_0_ for the hub node, *u*_1_ for uncontrolled periphery nodes, and *u*_2_ for a controlled periphery node.

To understand changes in optimal control strategies depending on predisposition strengths, we give an analytical argument for a star network and analyze two control scenarios: control of strength one focused at the central hub and control of strength one focused on a single peripheral node, cf. Figures 6A,B, respectively. Note that Masuda (2015) has also analyzed this toy network for the original voter model with a passive controller, finding that single node control should always be focused on the central hub in that case. As an illustrative example to investigate how the effects of control change with distance from a directly controlled node, we also investigate control of an undirected chain by placing a controller at one of the ends of the chain.

Our arguments below are based on applying equation (3) to the star network. With some algebraic manipulation (see Appendix B for a detailed derivation), for control of strengths *p*_0_ = 1 applied to the central hub, we obtain *u*_0_ = (1 − *q*)/(1 + *kq*(2 − *q*)) and *u*_1_ = (1 − *q*)*u*_0_ and thus

(8) $$X_{\text{central}}=\frac{k\left(1-q\right)+1}{k+1}\frac{1-q}{1+\mathrm{kq}\left(2-q\right)},$$

where *k* is the number of spoke nodes. For the periphery controlled scenario, similar calculations yield *u*_1_ = (1–*p*)*u*_0_, *u*_0_ = (1–*q*)/(*k*–(1–*q*)^2^(*k*–1))*u*_2_ and *u*_2_ = (1–*q*)/(2–(1–*q*)^2^/(*k*–1–*q*)^2^(*k*–1)) resulting in

(9) $$X_{\text{periphery}}=\frac{f+\left(1-q\right)\left(k-1\right)f+1}{k+1}\frac{1-q}{2-\left(1-q\right)f},$$

where

(10) $$f=\frac{1-q}{{\left(1-q\right)}^{2}+\mathrm{kq}\left(2-q\right)}.$$

Comparison of *X*_central(_*_q_*_)_ with *X*_periphery(_*_q_*_)_ reveals changes in the optimal strategy when *q* is increased, cf. Figure 6C, where we illustrate this scenario for *k* = 15 and observe that for low *q* hub control is optimal whereas for large *q* periphery control proves superior. To analyze what control strategy performs better depending on *q*, we first note that *X*_central_(*q* = 0) = *X*_periphery_(*q* = 0) = 1 and observe that ∂*X*_periphery_/∂*q*|*_q=_*_0_ = (1–2*k–4k*^2^)/(*k* + 1) whereas ∂*X*_central_/∂*q*|*_q_*_=0_ = (–1–4*k*–2*k*^2^)/(*k* + 1), i.e., for *k* ≥ 2 after starting at the same point for *q* = 0 the effectiveness of central control initially decays slower with *q* than the effectiveness of peripheral control. Thus, for small *q*, one has *X*_central_ > *X*_periphery_. As for both control scenarios *X*_central_(*q* = 1) > *X*_periphery_(*q* = 1) = 0, similar analysis of slopes at *q* = 1 shows that *X*_central_ > *X*_periphery_ for *q* close to 1.

Instead of a not very instructive exact calculation of the critical point *q*_crit_ at which optimal control switches, we limit the analysis to the case of large *k*. Figure 6C suggests that *q*_crit_ ≈ 1/2 for large *k* > 10 in star networks. In fact, expansion of equations (8) and (9) in leading order in 1/*k* confirms that *X*_central_ > *X*_periphery_ for *q* < 1/2 and *X*_central_ < *X*_periphery_ for q > 1/2 in the limit of *k* → ∞.

**Figure 7:**
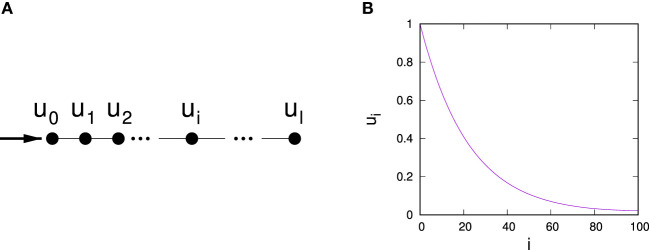
Illustration of a chain network with control targeted at node 0 at the left end **(A)** and dependence of the average stationary vote shares *u_i_* depending on the distance *i* to the directly controlled node **(B)** for a chain of length 100 and *q* = 0.01 calculated based on equation (19).

More importantly, calculations in this toy model illustrate why hub control weakens at large values of *q*. We note that for any *q* > 0, nodes are the more difficult to control the larger their degree. In fact, while this effect vanishes for *q* = 0, hub control also becomes the more difficult, the larger *q*. However, nodes are also the more difficult to (indirectly) control the farther away they are in terms of network distance from the node directly influenced by the controller. To analyze the latter effect, consider a linear chain of length *l*, controlled by influence of strength one applied to either end, cf. Figure 7A. Equation (3) applied to this situation then reads

(11) $$2u_{0}-\left(1-q\right)u_{1}=\left(1-q\right)$$

(12) ⋯

(13) $$\begin{array}{l} 2u_{i}-\left(1-q\right)u_{i-1}-\left(1-q\right)u_{i+1}=0\\ \cdots \\ u_{l}-\left(1-q\right)u_{l-1}=0. \end{array}$$

To solve the above system of linear homogeneous difference equations, we use the ansatz *u_i_* = *Aλ^i^* for *i* = 1, …, *n*–1 and find eigenvalues

(14) $$\lambda_{1/2}=\frac{1}{1-q}\left(1\pm g\right),$$

with $g=\sqrt{q\left(2-q\right)}$. General solutions are thus of the form $u_{i}=A\lambda_{1}^{i}+B\lambda_{2}^{i}$. Matching with the boundary conditions for *i* = 0 and *i* = 1 gives two conditions to fix the values of the constants *A* and *B*

(15) $$2\left(A+B\right)-\left(1-q\right)\left(A\lambda_{1}+B\lambda_{2}\right)=1-q$$

(16) $$A\left(\lambda_{1}^{l}+B\lambda_{2}^{l}\right)-\left(1-q\right)\left(A\lambda_{1}^{l-1}+B\lambda_{2}^{l-1}\right)=0.$$

Solving for *A* and *B* one obtains

(17) $$A=-B\frac{\lambda_{2}^{l-1}}{\lambda_{1}^{l-1}}\frac{g-1}{g+1}$$

and

(18) $$B=\frac{1-q}{1+{\left(\frac{1-g}{1+g}\right)}^{l+1}}.$$

We finally obtain

(19) $$u_{i}=\frac{1}{{\left(1-q\right)}^{i-1}}\frac{1}{1+{\left(\frac{1-g}{1+g}\right)}^{l+1}}\left(\frac{{\left(1-g\right)}^{l}}{{\left(1+g\right)}^{l}}{\left(1+g\right)}^{i}+{\left(1-g\right)}^{i}\right).$$

We observe that for *i* < *l*, the second term in equation (19) is always substantially larger than the first. Noting also that *q* < *g*(*q*) for *q* ∈ (0,1), it follows that *u_i_* is decreasing with *i*, i.e., the example of the controlled chain network demonstrates that influence of indirect control on a node decreases with the distance from that node, cf. also Figure 7B.

We thus see two opposing effects of hub control. On the one hand, hubs are the more difficult to control the larger their degree. On the other hand, because a hub node has more neighbors than an average node, control of hub nodes provides a controller with closer access to other nodes in the network, and this improved access can outweigh the enhanced difficulty of controlling high degree nodes for low predisposition strengths. In contrast, in high *q* settings, the decreased controllability of hub nodes outweighs the enhanced access to their respective neighbors that they provide to the controller.

## 4 Discussion

In this paper, we have investigated the impact of predispositions to return to the uninfluenced state on opinion control in a variant of the voter model. Results have shown that predisposition strength has a strong influence on optimal control strategies, such that essentially two control regimes exist. For low predisposition strength, optimal control is found to be focused on hub nodes, whereas for large predisposition strength, optimal control should be focused on low-degree nodes. In the latter situation, controllers can only gain relatively little total influence over the system, but strategic allocation can still result in improvements of control gains of up to 40% relative to random allocation.

Through numerical simulations of the voting dynamics on scale-free networks and analytical calculations on star networks, we have established that both regimes tend to be separated by a transition, with details of the transition depending on resource endowments of the controller and the heterogeneity of the social network. Our numerical results suggest that more heterogeneous networks (i.e., scale-free networks with a smaller scaling exponent *α*) support a larger regime of optimal hub control than more homogeneous networks.

Our main finding, i.e., the existence of regimes in which optimal control strategies should focus on low-degree nodes, differs markedly from previous investigations of the original voter model (Kuhlman et al., 2013; Yildiz et al., 2013; Masuda, 2015). A point that may serve to illustrate the reduced effectiveness of hub control in the present model is that the model can be mapped to the conventional voter model with a passive opponent who influences every voter on the social network. To account for the spontaneous state reversion, which occurs with constant probability for each node, in the mapped version of the standard voter model, such a passive controller would have to have control strengths to nodes proportional to their degree, i.e., exert a much stronger influence on hub nodes than low-degree nodes (see Appendix C for details). Thus, it is not surprising that a binary active controller may wish to focus on low-degree nodes. In this light, one might wonder why hub control is optimal for any value of *q*. As our toy example of a chain network has illustrated, indirectly controlling nodes that do not have a direct connection from the controller comes at a cost that grows with distance from the closest directly controlled node. Thus, hub nodes can still be optimal because of their central topological position in the network because of which average distances from them to other nodes are lower than for low-degree nodes, potentially outweighing the enhanced difficulty in gaining control over them.

In the presented model, we have analyzed the case of binary scenarios in which nodes can either be controlled or not, but controllers cannot choose the strengths of control. An alternative scenario could be an allocation scheme in which controllers can distribute resource in such a way that some nodes are strongly influenced and others only experience a weak effect. It is thus possible that our choice of binary control could have affected the results. One could imagine that even in the low-degree regime in the binary model, optimal continuous schemes that allocate very strong control to hubs could outperform evenly balanced control that aims to influence many low-degree nodes. Investigations of the continuous scenario represent an interesting avenue for future work.

Another point worth emphasizing is that we have considered undirected networks in this study. Results in the voter dynamics may differ markedly on directed networks (Masuda, 2015). Moreover, on directed networks, in- and out-degrees of nodes might be uncorrelated such that out-degree hubs are not necessarily in-degree hubs and vice versa. Difficulties in hub-control as described above relate to the difficulty of node control with growing in-degree, whereas benefits of node control result from large out-degrees. One can thus expect a more nuanced picture for directed networks in the presence of a predisposition to resist, which should be worth studying in more detail in the future.

On a more speculative note, we remark that predispositions to return to the uninfluenced state in the present model essentially introduce a degree-dependent resistance of nodes to align with the external control. A rewrite of equation (1) shows that a very similar equation and essentially similar effects can be observed when not introducing *q* as a probability to return to the opposed state when in the influenced state, but as a probability to flip state in *any* state. The latter phenomenon corresponds to noise, and it will be of interest to carry out a more detailed comparison of results for the voting dynamics in this situation with the results of Lynn and Lee (2016) for the kinetic Ising model.

## Author Contributions

MB designed the study, conducted and evaluated the experiments, and wrote the paper. All authors contributed to manuscript revision, read and approved the submitted version.

## Conflict of Interest Statement

The authors declare that the research was conducted in the absence of any commercial or financial relationships that could be construed as a potential conflict of interest.

## Appendix A

Here, we provide a more detailed derivation of equation (3). We start from equation (1), setting ${\dot{u}}_{i}=0$ to obtain a stationarity condition. One notes that the quadratic terms proportional to products *u_i_u_j_* cancel out and after multiplication by Σ*_i_*, we find

(A1) $$0=\left(1-q\right)\left(\sum_{j}a_{\mathrm{ji}}u_{j}+\left(1-u_{i}\right)p_{i}-u_{i}\sum_{j}a_{\mathrm{ji}}\right)-q\Sigma_{i}u_{i}.$$

Re-arranging terms and recalling $\Sigma_{i}=\sum_{j}\;a_{\mathrm{ji}}+p_{i}$ yields

(A2) $$-\left(1-q\right)p_{i}=\left(1-q\right)\sum_{j}a_{\mathrm{ji}}u_{j}-\left(1-q\right)u_{i}p_{i}-\left(1-q\right)u_{i}\sum_{j}a_{\mathrm{ji}}-qu_{i}\sum_{j}a_{\mathrm{ji}}-qp_{i}u_{i}$$

and thus

(A3) $$\left(1-q\right)p_{i}=u_{i}\Sigma_{i}-\left(1-q\right)\sum_{j}a_{\mathrm{ji}}u_{j},$$

which is equation (3).

## Appendix B

In this section, we provide some additional detail for the calculation of equilibrium shares for star networks with central and peripheral control (cf. Section 3.2 and Figure 6 for a pictorial representation of the corresponding networks). We start by analyzing central control, for which equation (3) reads

(A4) $$\left(k+1\right)u_{0}-\left(1-q\right)ku_{1}=\left(1-q\right)$$

(A5) $$u_{1}-\left(1-q\right)u_{0}=0.$$

From equation (A5), we find *u*_1_ = (1 − *q*)*u*_0_ and inserting into equation (A4) yields

(A6) $$u_{0}\left(\left(k+1\right)-k{\left(1-q\right)}^{2}\right)=\left(1-q\right).$$

We thus find the expression for *u*_0_ given in Section 3.2. We further have

(A7) $$X_{\text{central}}=\frac{1}{k+1}\left(u_{0}+ku_{1}\right)$$

(A8) $$X_{\text{central}}=\frac{u_{0}\left(1+k\left(1-q\right)\right)}{k+1},$$

which results in equation (8) in Section 3.2 after inserting *u*_0_.

We proceed with details of the calculation to obtain the controlled vote share for a periphery-controlled star. In this case, equation (3) reads:

(A9) $$ku_{0}-\left(1-q\right)\left(k-1\right)u_{1}-\left(1-q\right)u_{2}=0$$

(A10) $$u_{1}-\left(1-q\right)u_{0}=0$$

(A11) $$2u_{2}-\left(1-q\right)u_{0}=1-q.$$

We immediately see *u*_1_ = (1–*q*)*u*_0_. Inserting into equation (A9) and solving for *u*_2_ gives the expression in Section 3.2 for *u*_2_, which can be written in more convenient form using equation (10)

(A12) $$u_{2}=\frac{1-q}{2-\left(1-q\right)f}.$$

Finally, we have

(A13) $$X_{\text{periphery}}=\frac{1}{k+1}\left(u_{2}+fu_{2}+\left(k-1\right)\left(1-q\right)fu_{2}\right),$$

which, after inserting *u*_2_, results in expression (9) in Section 3.2.

## Appendix C

In this section, we provide a more detailed argument how the voting model with predisposition can be mapped to the original voter model in the presence of two opposing zealots, where the passive zealot has a control strength, which is proportional to a node’s degree. In the following, we shall label the active controller as A and its control influence as $p_{i}^{A}$ (as opposed to just labeling its control gain by *p_i_* in the rest of the paper), and label the passive controller as B with control gain $p_{i}^{B}$. We shall show that our equation (1) is equivalent to equation (3) in Masuda (2015) provided that the strength of the passive controller is proportional to the influence exerted on an agent by the social network and the active control, i.e., $p_{i}^{B}=\gamma \left(\sum_{j}\;a_{\mathrm{ji}}+p_{i}^{A}\right)$. We first note that we can scale time in our equation (1) to obtain an equivalent condition

(A14) $${\dot{u}}_{i}=\left(1-q\right)/\Sigma_{i}\left(\;\left(1-u_{i}\right)\left(\sum_{j}a_{\mathrm{ji}}u_{j}+p_{i}\right)-u_{i}\sum_{j}a_{\mathrm{ji}}\left(1-u_{j}\right)\right)-q/\left(1-q\right)u_{i},$$

We can now rewrite equation (A14) as

(A15) $${\dot{u}}_{i}=\frac{\sum_{j}\;a_{\mathrm{ij}}+p_{i}^{A}+p_{i}^{B}}{\sum_{j}\;a_{\mathrm{ji}}+p_{i}^{A}}\times \left(\left(1-u_{i}\right)\frac{\sum_{j}\;a_{\mathrm{ji}}u_{j}+p_{i}}{\sum_{j}\;a_{\mathrm{ji}}+p_{i}^{A}+p_{i}^{B}}-u_{i}\frac{\sum_{j}\;a_{\mathrm{ji}}\left(1-u_{j}\right)}{\sum_{j}\;a_{\mathrm{ji}}+p_{i}^{A}+p_{i}^{B}}\right)-\frac{q}{1-q}\frac{\sum_{j}\;a_{\mathrm{ij}}+p_{i}^{A}+p_{i}^{B}}{\sum_{j}\;a_{\mathrm{ij}}+p_{i}^{A}+p_{i}^{B}}u_{i},$$

and thus

(A16) $${\dot{u}}_{i}=\left(1+\gamma \right)\;\left(\;\left(1-u_{i}\right)\frac{\sum_{j}\;a_{\mathrm{ji}}u_{j}+p_{i}}{\sum_{j}\;a_{\mathrm{ji}}+p_{i}^{A}+p_{i}^{B}}-u_{i}\frac{\sum_{j}\;a_{\mathrm{ji}}\left(1-u_{j}\right)}{\sum_{j}\;a_{\mathrm{ji}}+p_{i}^{A}+p_{i}^{B}}\;\right)-\frac{q\left(1+\gamma \right)}{\left(1-q\right)\gamma }\frac{p_{i}^{B}}{\sum_{j}\;a_{\mathrm{ij}}+p_{i}^{A}+p_{i}^{B}}u_{i},$$

Again rescaling time by a factor (1 + *γ*), we find

(A17) $${\dot{u}}_{i}=\left(\left(1-u_{i}\right)\frac{\sum_{j}a_{\mathrm{ji}}u_{j}+p_{i}}{\sum_{j}a_{\mathrm{ji}}+p_{i}^{A}+p_{i}^{B}}-u_{i}\frac{\sum_{j}a_{\mathrm{ji}}\left(1-u_{j}\right)+q/\left(\gamma \left(1-q\right)\right)p_{i}^{B}}{\sum_{j}a_{\mathrm{ji}}+p_{i}^{A}+p_{i}^{B}}\right),$$

which corresponds to Masuda’s equation (3) provided we choose *γ* = *q*/(1–*q*). We thus see that the model with predisposition of strength *q* to resist influence is formally equivalent to the original controlled voter model with a passive controller who exerts a given degree-dependent control of strength $p_{i}^{B}=q/\left(1-q\right)\left(\sum_{j}\;a_{\mathrm{ji}}+p_{i}^{A}\right)$ on all nodes. As we consider binary control with $p_{i}^{A}\in \left\{0,1\right\}$, $p_{i}^{B}$ would have to be essentially proportional to degree for large degrees.
